# Abnormal iron metabolism in fibroblasts from a patient with the neurodegenerative disease hereditary ferritinopathy

**DOI:** 10.1186/1750-1326-5-50

**Published:** 2010-11-10

**Authors:** Ana G Barbeito, Thierry Levade, Marie B Delisle, Bernardino Ghetti, Ruben Vidal

**Affiliations:** 1 Department of Pathology and Laboratory Medicine and Indiana Alzheimer disease Center, Indiana University School of Medicine, 635 Barnhill Dr, MSB A136, Indianapolis, IN, 46202, USA; 2 Institut de Médecine Moléculaire de Rangueil, INSERM U.858, Université Toulouse III Paul-Sabatier, IFR31, Toulouse, France; 3 Service d'Anatomie et de Cytologie Pathologiques, Hopitaux de Toulouse, TSA 50032 - 31059 Toulouse, Cedex 4, France

## Abstract

**Background:**

Nucleotide duplications in exon 4 of the ferritin light polypeptide (FTL) gene cause the autosomal dominant neurodegenerative disease neuroferritinopathy or hereditary ferritinopathy (HF). Pathologic examination of patients with HF has shown abnormal ferritin and iron accumulation in neurons and glia in the central nervous system (CNS) as well as in cells of other organ systems, including skin fibroblasts. To gain some understanding on the molecular basis of HF, we characterized iron metabolism in primary cultures of human skin fibroblasts from an individual with the *FTL c.497_498dupTC *mutation.

**Results:**

Compared to normal controls, HF fibroblasts showed abnormal iron metabolism consisting of increased levels of ferritin polypeptides, divalent metal transporter 1, basal iron content and reactive oxygen species, and decreased levels of transferrin receptor-1 and IRE-IRP binding activity.

**Conclusions:**

Our data indicates that HF fibroblasts replicate the abnormal iron metabolism observed in the CNS of patients with HF. We propose that HF fibroblasts are a unique cellular model in which to study the role of abnormal iron metabolism in the pathogenesis of HF without artifacts derived from over-expression or lack of endogenous translational regulatory elements.

## Background

Abnormal brain iron metabolism leading to neurodegeneration is the main feature of diseases such as Friedreich ataxia (FRDA), aceruloplasminemia, neurodegeneration with brain iron accumulation type I (NBIA I), and hereditary ferritinopathy (HF) or neuroferritinopathy [1-3]. HF is an adult-onset autosomal dominant disease caused by nucleotide duplications in exon 4 of the ferritin light polypeptide (*FTL*) gene. Six different mutations have been reported, leading to an increase in the length and a change of the amino acid sequence of the C-terminus of FTL [4-9]. HF affects the central nervous system (CNS) presenting clinically as an extra-pyramidal movement disorder accompanied by cognitive and behavioral disturbances, starting between the third and sixth decade of life [10]. Neuropathologically, HF is characterized by a severe neuronal loss in the basal ganglia, atrophy of cerebellum and cerebral cortex, abnormal iron accumulation, and the presence of ferritin inclusion bodies (IBs) in neurons and glia [3]. Ferritin IBs are not limited to the CNS since they can also be seen in hepatocytes, cells of the renal tubular epithelium, endothelial cells of capillaries, and skin fibroblasts [5,6].

Ferritin is the main intracellular iron storage protein, having a central role in the regulation of cellular iron metabolism and iron detoxification [11,12]. Mammalian ferritin consists of 24 subunits of FTLs and ferritin heavy polypeptides (FTH); the FTH subunit is involved in the rapid detoxification of iron, whereas the FTL subunit facilitates iron nucleation, mineralization, and long-term iron storage [13]. Ferritin provides both a source of metabolic active iron and also serves as an oxygen free radical cytoprotective protein, storing iron that is not needed for immediate metabolic use [11,12]. Each subunit consists of a bundle of 4 parallel α-helices (A, B, C, and D), a long extended loop (connecting helices B and C), and a C-terminus with a short α-helix (E) which is involved in important stabilizing interactions around the 4-fold symmetry axes [12]. Spectroscopic and biochemical studies of recombinant mutant FTL homopolymers assembled from the p.Phe167SerfsX26 polypeptide (originated from the *c.497_498dupTC *mutation) [5] have shown that the mutation causes conformational changes in ferritin, altering iron incorporation and promoting iron-mediated aggregation of ferritin. The process of iron-induced aggregation of ferritin does not seem to involve covalent bonds since it can be reversed by iron-chelants both *in vitro *and *in vivo *[14]. X-ray crystallographic analysis of homopolymers of the mutant p.Phe167SerfsX26 polypeptide showed the complete absence of the E helical domain of FTL in mutant subunits and substantial disruption of the 4-fold pores of the 24-mer [15]. Transgenic expression of the p.Phe167SerfsX26 polypeptide in mice recapitulated several features of the disease, including intracellular formation of ferritin IBs in neurons and glia in the CNS and in cells of other organ systems, including skin fibroblasts [16]. Transgenic mice showed dysregulation of iron homeostasis and evidence of oxidative damage in the brain, similarly to what has been observed in individuals with HF [17]. Herein, we report ferritin accumulation, iron dyshomeostasis and evidence of oxidative stress in human skin fibroblasts from a patient with HF. Our results reveal that the broad dysfunction of iron homeostasis observed in individuals with HF and in the transgenic animal model of HF is replicated in HF skin fibroblasts. We propose that HF skin fibroblasts represent a unique cellular model in which to study the molecular mechanisms of cellular toxicity that may lead to neurodegeneration in HF.

## Results

### Ferritin polypeptides accumulate in skin fibroblasts expressing mutant FTL

**Figure 1:**
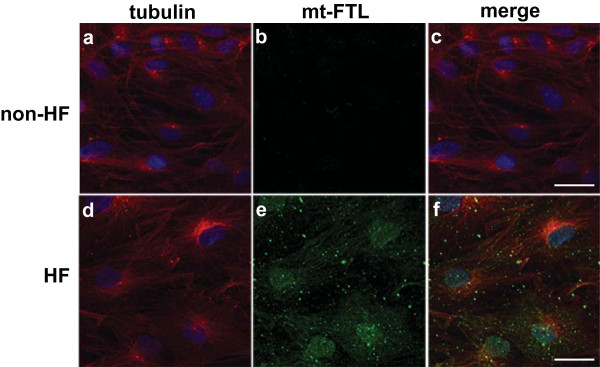
**Accumulation of ferritin in cultured HF skin fibroblasts**. Confocal immunofluorescence microscopy was performed on cultured confluent wild-type (non-HF) (a-c) and HF (d-f) fibroblasts using antibodies against alpha-tubulin (a, d) and mutant FTL (b, e). Nuclei were stained by DAPI (blue). A merge of the alpha-tubulin (red) and mutant FTL (green) images is shown in c and f. Mutant FTL was observed only in HF fibroblasts, both in the cytoplasm and nucleus. Scale bars: 30 μm.

**Figure 2:**
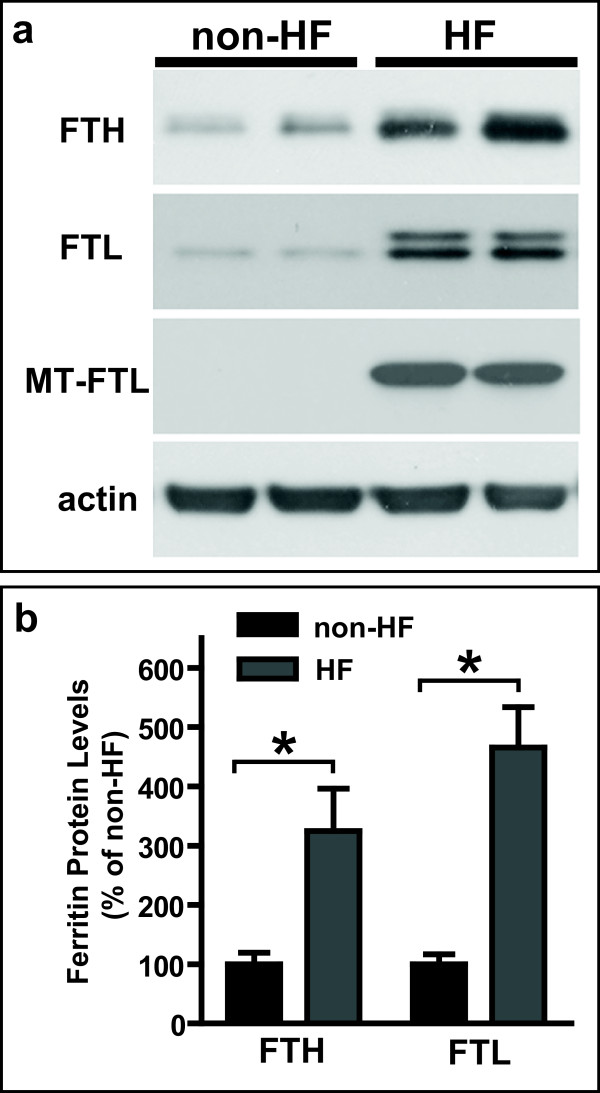
**Increased levels of ferritin polypeptides in HF skin fibroblasts**. a) Representative western blot showing the expression of ferritin subunits in non-HF and HF fibroblasts. Western blot was carried out using antibodies against ferritin heavy chain (FTH), the amino terminus of ferritin light chain (FTL) that recognizes wild-type and mutant FTL subunits, and abs specific for the mutant subunit (MT-FTL). b) Densitometric analysis of ferritin subunit protein levels shows increased levels of FTH and wild-type and mutant FTL. Results are the means and SD of three independent experiments (*p < 0.05).

Confocal fluorescence microscopy analysis (Figure 1) using abs against the mutant-FTL chain showed that the mutant polypeptide accumulated both in the cytoplasm and in the nucleus of HF fibroblasts (Figure 1e), as previously reported in skin fibroblasts of individuals with HF and transgenic mice [5,16]. No staining was observed in normal control skin fibroblasts (non-HF) (Figure 1b). Western blot analysis showed expression of the ferritin subunits in normal and in HF fibroblasts (Figure 2a). Antibodies against the N-terminus of FTL detected two bands on western blots of HF fibroblasts, in agreement with the difference in molecular weight between wild-type and mutant-FTL polypeptides [5]. Densitometric analysis showed a statistically significant (p < 0.05) increase in the levels of ferritin light and heavy polypeptides in HF fibroblasts (FTH1: 3.2 fold; FTL: 4.7 fold) (Figure 2b).

### Altered expression of proteins of iron metabolism in HF fibroblasts

**Figure 3:**
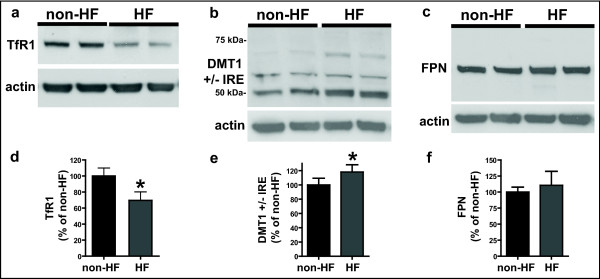
**Expression of proteins of iron metabolism**. Representative western blots showing the expression of: a) TfR-1, b) DMT1 (+/-) IRE, and c) FPN, in non-HF and HF fibroblasts. Results are the means and SD of three independent experiments. Densitometric analysis shows a significant decrease in TfR1 levels (d) and a significant increase in total levels of DMT1 isoforms (e) in HF compared to non-HF fibroblasts.(*p < 0.05). No significant changes in the levels of FPN (f) were observed.

Western blot analysis of the expression of TfR1 and DMT1 + and - IRE isoforms (+/-IRE-DMT1) showed significant changes in the levels of both proteins (Figure 3a, b). Densitometric analysis showed a statistically significant (p < 0.05) decrease in the levels of TfR1 (reaching a level of 70 ± 11% of non-HF control) and a statistically significant (p < 0.05) increase in the total levels of the iron influx transporter +/-IRE-DMT1 (118 ± 10% of non-HF control), mainly in bands corresponding to molecular weights of ~70 kDa and ~50 kDa. No significant changes were observed for FPN (Figure 3c, f).

### Cellular iron status in HF fibroblasts

**Figure 4:**
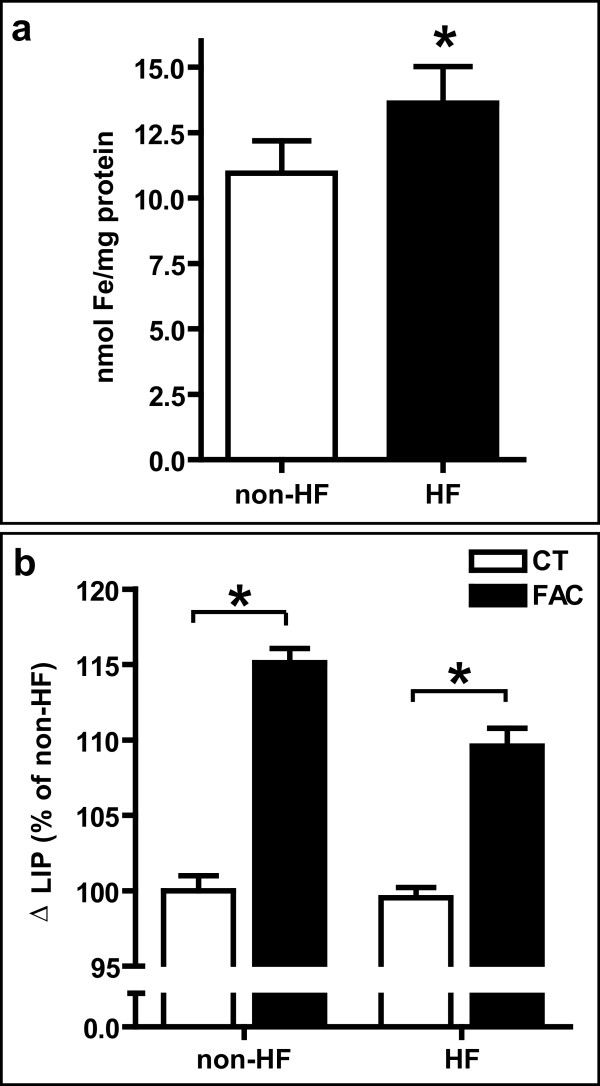
**Cellular iron levels**. a) Fibroblasts were cultured under basal conditions (BC) and total iron content was analyzed by the ferrozine method. A statistically significant difference (*p < 0.05) in the levels of iron content (nmol Fe/mg protein) was observed in HF compared to non-HF fibroblasts. b) Analysis of the labile iron pool (LIP) in basal conditions and after treatment with 100 μM FAC for 72 h. The LIP was measured using the metal-sensitive fluorescence probe calcein. No statistically significant differences were observed under basal conditions. After FAC treatment, LIP levels increased significantly in both non-HF and HF fibroblasts. However, FAC-induced levels of LIP were significantly lower in HF compared to non-HF fibroblasts. *p < 0.05

Total iron contents were measured using a ferrozine-based iron assay [18]. Mutant fibroblasts showed a statistically significant (p < 0.05) increase in the levels of basal iron content when compared to non-HF fibroblasts (non-HF: 10.9 ± 1.2 nmol Fe/mg protein; HF: 13.6 ± 1.4 nmol Fe/mg protein) (Figure 4a). LIP, the iron available for metabolism processes in the cell, was measured using a modified calcein assay [19-21]. No statistically significant differences in the levels of LIP were observed under basal conditions (BC) between HF and non-HF fibroblasts. LIP levels in normal and HF fibroblasts increased with statistical significance upon exposure to 100 μM FAC (Figure 4b).

### IRE-IRP binding activity

**Figure 5:**
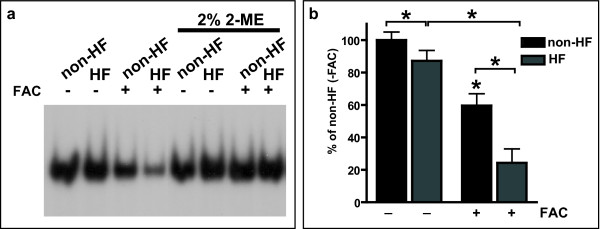
**IRE-IRP binding activity is decreased in HF skin fibroblasts**. a) Cytoplasmic extracts from non-HF and HF fibroblasts were analyzed for IRE-IRP binding activity using a gel-shift assay with a ^32^P-labelled RNA probe containing a ferritin IRE sequence in the absence or presence of 2% 2-mercaptoethanol (2% 2-ME) to promote maximal IRE-IRP1 binding. Fibroblasts were cultured in the presence (+) or absence (-) of 100 μM ferric ammonium citrate (FAC) for 72 h. A lower binding activity of IRP1 was observed under basal conditions (no FAC) in HF fibroblasts. After FAC treatment, HF fibroblasts showed significantly less binding activity than controls (HF: 30 ± 7%; non-HF: 62 ± 4%). *p < 0.05.

Under basal conditions, IRE-IRP binding was found to be reduced in HF fibroblasts when compared to normal fibroblasts (non-HF: 100 ± 12%; HF: 88 ± 8%) (Figure 5). When fibroblasts where subjected to FAC treatment for 72 h, a significant decrease in IRE-IRP binding was observed in HF and non-HF fibroblasts. Interestingly, the change in IRE-IRP binding after treatment with FAC was more pronounced in HF fibroblasts than in control fibroblasts (non-HF: 60 ± 7%; HF: 24 ± 9%) (Figure 5b). After 2-ME treatment to promote maximal IRE-IRP1 binding [22,23], IRP binding activity increased nearly to 100%, suggesting that the majority of the observed decreased in binding activity may be associated with binding of IRP1 rather than IRP2 [22,23].

### Oxidative damage in HF fibroblasts

**Figure 6:**
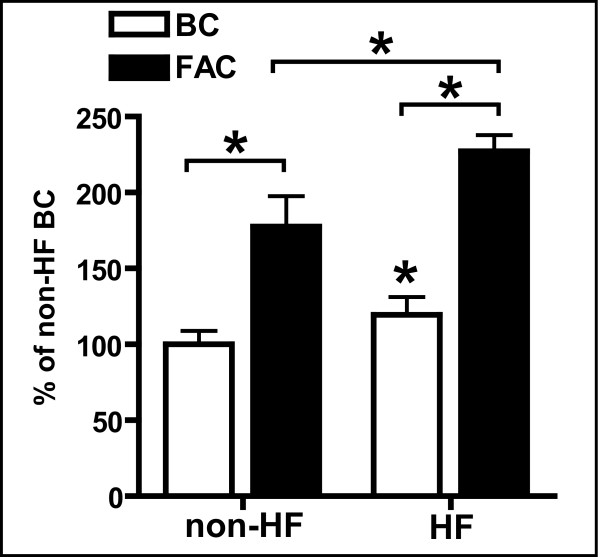
**Oxidative stress in HF skin fibroblasts**. Fibroblasts were grown in standard culture medium and then cultured for 72 h in medium containing 100 μM FAC. Cells were subsequently loaded with DHCF-DA and DCF fluorescence was determined. Significantly higher levels of ROS were observed in HF fibroblasts under basal conditions. After FAC treatment, HF fibroblasts showed significantly higher levels of ROS than wild-type fibroblasts. *p < 0.05.

ROS formation in cultured fibroblasts was determined using the fluorescent dye 2',7'-dichlorodihydrofluorescein under basal conditions and after treatment with FAC. Under basal conditions, HF fibroblasts showed significant higher levels of ROS than non-HF fibroblasts (Figure 6). A statistically significant increase in cellular oxidant levels was observed in normal and HF fibroblasts after loading with FAC for 72 h. Interestingly, ROS levels were significantly higher in HF fibroblasts compared to non-HF fibroblasts after FAC treatment.

## Discussion

The neurodegenerative disease neuroferritinopathy or HF is an autosomal dominant, adult onset disease caused by mutations in the *FTL *gene that lead to the production of FTL polypeptides with abnormal C-termini [3-9,24]. The clinical presentation of HF varies both within and between families. Patients may present with tremor, cerebellar signs, parkinsonism, psychiatric problems, abnormal involuntary movements (dystonia, chorea), pyramidal syndrome, pseudo-bulbar symptoms, and cognitive deficit [3-10]. Magnetic resonance imaging shows abnormal signals in the globus pallidus and putamen, and cavitation in the putamen [5,10]. Mild cerebral and cerebellar atrophy may be observed. Serum ferritin levels may be within normal range or even decreased in some patients [4]. Neuropathologic studies show cavitation in the putamen [4-6]. The cerebrum and cerebellum are atrophic. Ferritin IBs are found in nuclei and cytoplasm of glial cells and neurons in the CNS as well as in cells of other organ systems and are labeled by antibodies against light and heavy chains of ferritin and antibodies specific for the mutant FTL polypeptide [5]. Abnormal iron accumulation (both as ferrous and ferric iron) associated with ferritin IBs has been described [4-6] as well as evidence of oxidative damage in patients [6] and in animal models [17,25]. Thus, disrupted iron homeostasis and iron-mediated oxidative stress may have a major role in the pathogenesis of HF [3,24].

**Figure 7:**
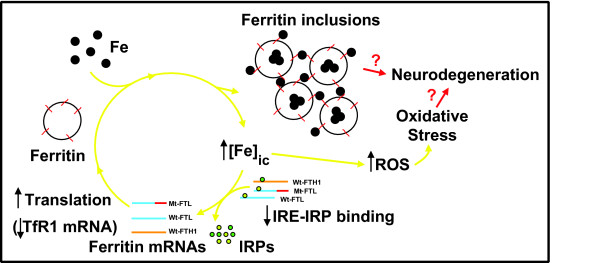
**Iron-induced ferritin aggregation and oxidative stress in HF**. The mutant FTL polypeptide acts as a dominant negative mutant, leading to a failure of ferritin in its iron storage function and an increase in the levels of intracellular (ic) iron, resulting in the release of the IRP proteins from the ferritin IRE and degradation of *TfR1 *mRNA, generating a positive feed-back loop that leads to over-production of ferritin polypeptides. The C-termini of the mutant FTL polypeptides may extend above the spherical shell allowing them to crosslink with other ferritin molecules through iron bridging, promoting iron-mediated aggregation of ferritin. Free iron leads to the generation of ROS and oxidative stress. Ferritin aggregates and oxidative stress may lead to neurodegeneration in HF.

We investigated cellular iron metabolism in primary cell cultures of skin fibroblasts from an individual with the *c.497_498dupTC *mutation to determine whether fibroblasts would be an accessible *in vitro *system in which to directly examine disease-relevant pathologic mechanisms in HF. Our results clearly show alterations of iron metabolism in HF fibroblasts, which seem to recapitulate the abnormalities seen in HF patients and transgenic mice [5,6,16,17]. Ferritin is considered a cytoplasmic iron-storage protein; however, a nuclear localization for ferritin [26] has been described in animal models of iron overload [27], and in avian [28] and human astrocytoma cells [29]. In HF fibroblasts, we observed ferritin accumulation in the nucleus and cytoplasm of the cells under basal conditions, as reported in cells from patients affected by HF [5] and in transgenic mice over-expressing mutant FTL [16]. Ferritin accumulation may be explained by the formation of ferritin aggregates and/or by overproduction of ferritin (enhanced transcription/translation of ferritin mRNAs) by the cells in response to a diminished iron-binding activity of mutation-containing ferritin. We have previously shown that ferritin containing the mutant FTL polypeptide has a diminished ability to sequester and store mineralized iron *in vitro *[14,15,30] and does not lead to an increase in the transcription of ferritin polypeptide genes *in vivo *in mice, although transgenic mice accumulate ferritin as intracytoplasmic and intranuclear IBs [16,17]. Thus, we proposed that the mutant FTL polypeptide may act *in vivo *as a dominant negative mutant, causing the failure of ferritin in its iron storage function and leading to an increase in the levels of intracellular iron and the translation of ferritin mRNAs (Figure 7) [3]. In HF fibroblasts, we observed an increase in protein levels of ferritin polypeptides and a decrease in the protein levels of TfR1. Proteins of iron metabolism are known to be regulated in a coordinated fashion mainly at the post-transcriptional level [31]. IRPs when interacting with IRE at the 5' -untranslated regions (UTR) of *FTH1 *and *FTL *mRNA inhibit their translation, whereas the interaction between IRPs with the IREs located at the 3' UTR of *TfR1 *mRNA stabilizes the mRNA, increasing TfR1 protein levels [32]. Thus, the finding of increased protein levels of FTL and FTH and a decrease in TfR1 protein levels in concert with a decrease in the binding activity of IRP-IRE in HF fibroblasts strongly supports the notion of a failure of ferritin in its iron storage function in HF fibroblasts, leading to an increase in *FTL *and *FTH1 *translation and degradation of *TfR1 *mRNA. As would be expected, treatment with FAC of normal and HF fibroblasts leads to a significant decrease in IRE-IRP binding in both cases. However, the change in IRE-IRP binding after treatment with FAC was more pronounced in HF fibroblasts than in control fibroblasts, suggesting a lower IRE-IRP binding activity and consequent translation of ferritin mRNA and destabilization of *TfR1 *mRNA in HF fibroblasts. In agreement with the data obtained in transgenic mice, no significant changes in the protein levels of the iron exporter FPN were observed [17]. We detected an increase in the protein levels of DMT1 (+/-IRE) in HF fibroblasts, which may account for the increase in total iron observed in HF fibroblasts. DMT1, a proton-coupled metal transporter that participates in iron influx to the cell from the extracellular matrix and/or from recycling endosomes [33] has been shown to contribute to the neurodegenerative process occurring in Parkinson disease (PD), where an increase in the expression of DMT1 (+IRE) in the sustancia nigra of patients with PD correlates with an increase in iron content [34].

The expression profile of iron related-proteins and the IRE-IRP binding activity in HF fibroblasts suggest that the cells might be exposed to elevated levels of intracellular iron. Indeed, we observed that the total cellular iron levels were increased in HF fibroblasts compared to wild-type fibroblasts under basal conditions, although no statistically significant differences between control and HF fibroblast were observed in the levels of the iron available for metabolic processes in the cell (or LIP) under basal conditions. LIP in quiescent conditions represents only a minor fraction of the total cell iron and can undergo dynamic changes in response to various treatments [35], as seen when HF and non-HF fibroblasts were challenged with iron. The observed response of LIP to iron treatment suggests differential management of free intracellular iron in HF fibroblasts. HF fibroblasts showed increased ROS production compared to non-HF fibroblasts, which is in agreement with the finding of an increase in iron levels in HF fibroblasts under basal conditions. Iron imbalance and augmented cellular sensitivity to oxidative damage has also been observed in HeLa cells over-expressing the mutant ferritin *c.460dupA or the c.497_498dupTC *[36]. These data are consistent with studies in transgenic mice, which showed increased iron levels, lipid peroxidation, protein carbonylation, and nitrone products in the CNS [17], and the biochemical and immunohistochemical evidence for oxidative damage, lipid peroxidation and abnormal protein nitration observed in individuals with HF [6].

## Conclusions

The abnormally high iron levels observed in several neurodegenerative disorders have been proposed to lead to oxidative stress and neurodegeneration [17,36-39]. In HF, we found that a defect in a protein of iron metabolism leads to neurodegeneration, directly connecting abnormal iron metabolism and neurodegeneration (Figure 7). Our findings suggest that HF fibroblasts recapitulate the abnormal iron metabolism seen in patients with HF and in a transgenic animal model of HF. Further studies using the HF fibroblast cell model will provide additional insights into the mechanism(s) by which dysfunction of iron homeostasis may lead to neurodegeneration. In addition, they may be important in the characterization of therapeutic agents for HF and related diseases.

## Methods

### Materials

Dulbecco's Minimal Essential Medium (DMEM) 4.5 g/L glucose, fetal bovine serum (FBS), L-glutamine, penicillin/streptomycin, trypsin-EDTA solution and phosphate buffered saline (PBS) were obtained from Invitrogen (Carlsbad, CA). All cell culture plastics were obtained from Nunc (Rochester, NY, USA). All other general laboratory chemicals were obtained from Sigma-Aldrich Corp. (St. Louis, MO), unless specified.

### Fibroblast Cell Culture

Adult skin fibroblasts were isolated from an individual with the *c.497_498dupTC *mutation, which has been reported in members of a French family with HF [5]. Normal adult human skin fibroblasts were purchased from PromoCell GmbH (Heidelberg, Germany). Fibroblasts were cultured in DMEM supplemented with 10% FBS, 2.0 mM L-glutamine, and 100U/ml penicillin/100 μg/ml streptomycin, and incubated at 37°C in humidified air with 5% CO_2_. Cells from trypsinized monolayers were seeded at 8-10 × 10^3 ^cells/cm^2 ^and grown to confluence. For treatment with ferric ammonium citrate (FAC), confluent cells were incubated for 72 h with 100 μM FAC in DMEM in the absence of serum.

### Western blot analysis

Confluent cell cultures were lysed in tris buffered saline (TBS) containing 1% sodium dodecyl sulfate (SDS) and protease inhibitors (PI, Complete, Roche, Indianapolis, IN). Protein extracts (30 μg) were run in 4-20% SDS-polyacrylamide gels (Pierce, Rockford, IL, USA) and transferred to nitrocellulose membranes (GE Healthcare, Piscataway, NJ, USA). Membranes were blocked for 1 h in TBS containing 0.1% Tween-20 and 5% non-fat dry milk, followed by an overnight incubation with primary antibody (ab). After washing in TBS containing 0.1% Tween-20, the membranes were incubated with peroxidase-conjugated secondary ab (1:5000; GE Healthcare) for 1 h. Membranes were developed using the ECL chemiluminescent detection system (GE Healthcare). Densitometric analyses were performed using the NIH ImageJ program and normalized against the signal obtained by re-probing the membranes with anti-β-actin abs (Sigma-Aldrich). Primary antibodies were: anti-Transferrin Receptor-1 (TfR1; 1:500; Zymed Laboratories, South San Francisco, CA), anti-divalent metal transporter-1 (DMT1, recognizing both +/- IRE isoforms; 1:250; Alpha Diagnostic International, San Antonio, TX), anti-ferroportin-1 (FPN; 1:250; Alpha Diagnostic International), anti-FTL (D-18, 1:250, Santa Cruz Biotechnology, Santa Cruz, CA), anti-mutant FTL (ab1283, 1:8000) that recognizes specifically the human mutant FTL chain [5], anti-FTH (1:2000; Abcam, Cambridge, MA), and anti β-actin (1:10000; Sigma, St. Louis, MO). Nomenclature of the proteins is according to http://BioIron.org.

### Immunocytochemistry

Cultured fibroblasts were fixed in PBS + 4% paraformaldehyde (pH 7.4), at 4°C for 15 min and washed three times with PBS. Cells were permeabilized and blocked in 10% goat serum (Invitrogen), 2% bovine serum albumin (BSA) and 0.1% Triton X-100 in PBS and incubated with ab1283 (1:100) and mouse anti-α-tubulin (1:500; Sigma, St. Louis, MO). Secondary abs were: Alexa-Fluor488 goat anti-rabbit and Alexa-Fluor594 goat anti-mouse (Molecular Probes Inc, Eugene, OR). Fluorescence images were acquired with a Zeiss LSM 510 confocal microscope using an inverted 40× NA 1.2 objective.

### Electromobility shift assay

Iron response element (IRE)-binding activity was analyzed by gel retardation assay as described [22,23]. Briefly, cultured confluent fibroblasts were trypsinized and homogenized in extraction buffer (10 mM Hepes, pH 7.5, 3 mM MgCl_2_, 40 mM NaCl, 5% glycerol, 1 mM dithiothreitol, and 0.2% Nonidet P-40) at 4°C. After lysis, samples were centrifuged for 2 min at 10,000 *X g *to remove nuclei. The cytoplasmic extracts were diluted to a protein concentration of 3 mg/mL in lysis buffer without Nonidet P-40. The RNA IRE probe was transcribed in vitro from a linearized plasmid template (kindly provided by Dr. K. Pantopoulos) using T7 RNA polymerase in the presence of [α-^32^P]UTP as described [23]. For the RNA-protein complexes, 25 μg of cytoplasmic extract was incubated at room temperature with the radiolabeled ferritin IRE probe (25,000 cpm) in the absence or presence of 2% 2-mercaptoethanol (2-ME). After 10 min, heparin (50 μg) was added to the reaction to inhibit non-specific protein interactions with the probe and the incubation was continued for another 10 min. Unprotected probe was degraded by incubation with 1 U of RNase T1 for 10 min. RNA-protein complexes were analyzed in 6% non-denaturing polyacrylamide gels and visualized by autoradiography. Bands were scanned and the ratios of the intensity of the IRE-IRP complexes formed in the absence or presence of 2-ME were determined.

### Quantitation of intracellular iron by the colorimetric ferrozine assay

Total iron content was determined as previously described [18] with minor modifications. Briefly, cultured fibroblasts plated on 60 mm dishes were lysed in 0.9 ml of 50 mM NaOH for 2 h on a shaker in a humidified atmosphere. A standard curve was set using FAC standards (0-40 μM) in 10 mM HCl. Aliquots of cell lysates or standards were placed in 1.5 mL tubes and mixed with HCl or NaOH, respectively, to reach the same final concentration. Iron-releasing reagent (1.4M HCl:4.5% (w/v) KMnO_4 _in H_2_O) was added and the mixture incubated for 2 h at 60°C, in order to release iron-containing proteins. After cooling to room temperature, 60 μL of the iron-detection reagent (6.5 mM ferrozine, 6.5 mM neocuproine, 2.5 M ammonium acetate, and 1 M ascorbic acid dissolved in H_2_O) was added to each tube. After 30 min, 280 μL of the solution in each tube was transferred into a well of a 96-well plate and the absorbance was measured at 570 nm on a BioTek EL800 microplate reader (BioTek Instruments, Inc., Winooski, VT). The intracellular iron concentration was normalized against the protein content of the sample.

### Determination of the Labile Iron Pool (LIP)

LIP was measured using the metal-sensitive fluorescence probe calcein [19,20] in attached cells as described [21] with minor modifications. Briefly, confluent cell monolayers were first treated for 72 h in the presence of vehicle or 100 μM FAC. The cells were washed twice with PBS and loaded with calcein-acetoxymethyl ester (Invitrogen) at a final concentration of 0.25 μM for 15 min at 37°C (from a 1 mM stock solution in dimethyl sulfoxide) in loading medium (DMEM without sodium bicarbonate, 20 mM Hepes pH 7.4, 1 mg/ml BSA). Cells were washed and loaded in measuring media (20 mM Hepes pH 7.4, 150 mM NaCl, 5 mM glucose). After reaching baseline (3 min) first fluorescence readings (F1) were taken using a Beckman DTX880 fluorimeter (Beckman Coulter Inc., Fullerton, CA) in bottom read technique with a fluorescein optical filter (excitation: 485 nm; emission: 535 nm). Finally, the cells were depleted of iron in the presence of the membrane-permeable iron chelator salicylaldehyde isonicotinoyl hydrazone (SIH), kindly provided by Dr. Prem Ponka [40] and a second measurement of fluorescence was performed (F2) after 20 min. The F2/F1 ratio was used as a relative indicator of LIP independent of the cell number and distribution within the wells.

### Quantification of reactive oxygen species (ROS)

ROS formation was quantified by measuring fluorescence intensity of cultures after loading cells with 5-(and-6)-chloromethyl-2',7'-dichlorodihydrofluorescein diacetate (CM-H_2_DCFDA; Molecular Probes) [41]. CM-H_2_DCFDA, a cell-permeable indicator for reactive oxygen species, is hydrolyzed inside cells to a non-fluorescent compound which emits fluorescence when oxidized. Cultures were washed with Hank's Balanced Salt Solution (HBSS) containing calcium and magnesium. 10 μM DCFH-DA was loaded and incubated for 15 min at 37°C. Cultures were then washed in HBSS and fluorescence measurements were taken at 37°C using a Beckman DTX880 fluorimeter in bottom read technique with a fluorescein optical filter (excitation: 485 nm; emission: 535 nm). Background fluorescence from cultures subjected to sham dye incubation was subtracted from each recording.

### Statistics

All data are presented as the mean ± SD. Samples were run on duplicate or triplicate and each experiment was repeated at least three times. Statistical analysis was performed using the Student's t-test or a one-way analysis of variance followed by the Student's-Newman-Keuls test for multiple comparisons using GraphPad Prism version 4.03 (GraphPad Software Inc, La Jolla, CA). Differences were declared statistically significant if p < 0.05. When normality test failed, comparison of the means was performed by Weltch test.

## Competing interests

The authors state that there is no actual or potential conflicts of interest including any financial, personal or other relationships with other people or organizations within five years of beginning the work submitted that could inappropriately influence (bias) their work. The authors do not have any non-financial competing interests (political, personal, religious, ideological, academic, intellectual, commercial or any other) to declare in relation to this manuscript.

## Authors' contributions

AGB carried out experiments and analyzed data. TL and MBD extracted patients' fibroblasts and propagated them. BG critically read through the paper. AGB and RV designed the experiments and wrote the paper with assistance from all other authors. All authors read and approved the final manuscript.
